# Association of terpinolene and diclofenac presents antinociceptive and anti-inflammatory synergistic effects in a model of chronic inflammation

**DOI:** 10.1590/1414-431X20165103

**Published:** 2016-06-20

**Authors:** E.M.A. Macedo, W.C. Santos, B.P. Sousa, E.M. Lopes, C.A. Piauilino, F.V.M. Cunha, D.P. Sousa, F.A. Oliveira, F.R.C. Almeida

**Affiliations:** 1 Núcleo de Pesquisas em Plantas Medicinais, Universidade Federal do Piauí, Teresina, PI, Brasil; 2 Departamento de Ciências Farmacêuticas, Universidade Federal da Paraíba, João Pessoa, PB, Brasil

**Keywords:** Terpinolene, Inflammation, Antinociception, Pharmacological synergism

## Abstract

Pharmacological treatment of inflammatory pain is usually done by administration of non-steroidal anti-inflammatory drugs (NSAIDs). These drugs present high efficacy, although side effects are common, especially gastrointestinal lesions. One of the pharmacological strategies to minimize such effects is the combination of drugs and natural products with synergistic analgesic effect. The monoterpene terpinolene (TPL) is a chemical constituent of essential oils present in many plant species, which have pharmacological activities, such as analgesic and anti-inflammatory. The association of ineffective doses of TPL and diclofenac (DCF) (3.125 and 1.25 mg/kg *po*, respectively) presented antinociceptive and anti-inflammatory effects in the acute (0, 1, 2, 3, 4, 5 and 6 h, after treatment) and chronic (10 days) inflammatory hyperalgesia induced by Freund's complete adjuvant (CFA) in the right hind paw of female Wistar rats (170-230 g, n=6-8). The mechanical hyperalgesia was assessed by the Randall Selitto paw pressure test, which determines the paw withdrawal thresholds. The development of edema was quantified by measuring the volume of the hind paw by plethismography. The TPL/DCF association reduced neutrophils, macrophages and lymphocytes in the histological analysis of the paw, following a standard staining protocol with hematoxylin and eosin and the counts were performed with the aid of optical microscopy after chronic oral administration of these drugs. Moreover, the TPL/DCF association did not induce macroscopic gastric lesions. A possible mechanism of action of the analgesic effect is the involvement of 5-HT2A serotonin receptors, because ketanserin completely reversed the antinociceptive effect of the TPL/DCF association. These results suggest that the TPL/DCF association had a synergistic anti-inflammatory and analgesic effect without causing apparent gastric injury, and that the serotonergic system may be involved in the antinociceptive effect of this association.

## Introduction

**Figure 1:**
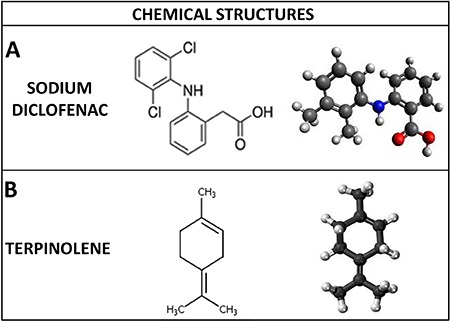
*A*, Sodium diclofenac. *B*, Monoterpene terpinolene (4-isopropylidene-1- methylcyclohexene) (Opdyke, 1988).

Inflammatory pain is characterized by an increased sensitivity of the injured tissue generated by the release of inflammatory mediators (1). Pharmacological treatment of inflammatory pain is usually performed with nonsteroidal anti-inflammatory drugs (NSAIDs), which show high efficacy (2). The sodium diclofenac (DCF) (Figure 1A) is a NSAID that acts by cyclooxygenase (COX) 1 and 2 inhibition, and it has been available in the medical arsenal for over 40 years, ranked as the eighth most sold drug in the world (3). However, about 20% of individuals experience side effects during treatment with NSAIDs, including abdominal pain, heartburn and diarrhea (4).

The monoterpenes are extracted from several herbs and represent 90% of the essential oils constituents (5). In relation to its pharmacological effects, 27 and 32 monoterpenes were found to have analgesic (6) and anti-inflammatory activities (7), respectively.

Given the difficulty of finding an effective drug with minimal side effects for the treatment of pain syndromes, researchers have been looking for other pharmacological strategies, such as drug combinations. There are studies showing that the combination of NSAIDs with monoterpenes have synergistic analgesic and anti-inflammatory effects with fewer gastric side effects (8,9).

This study evaluated the effect of the monoterpene terpinolene (TPL) (4-isopropylidene-1-methylcyclohexene) (Figure 1B) associated with DCF in acute and chronic inflammatory hyperalgesia. This monoterpene is found in the most diverse regions of the world in plant species, such as *Melaleuca alternifolia* C. - Australia (10); *Pistacia vera* L. - Greece, India and Iran (11); *Artemisia dracunculus* L. - Iran (12); and *Rosmarinus officinalis* L. - Brazil (13).

There are few studies on the TPL pharmacological activity, such as its antioxidant activity (14), or as one of the major components of the essential oils that showed anti-inflammatory (15), antibacterial (16), and anticancer (17) actions.

Based on the described benefits of NSAIDs, herbal drugs, or their combination, we evaluated the effect of the TPL, DCF and the association of both (TPL/DCF) on the complete Freund's adjuvant (CFA)-induced inflammatory process, as well as the incidence of gastric damage.

## Material and Methods

### Drugs and chemicals

The following substances were used: TPL (Sigma, USA), CFA (Difco, USA), DCF (Galen, Brazil), and ketanserin (Sigma). For the pharmacological studies, the TPL was suspended in 2% Tween 80 in 0.9% saline (10 mL/kg). The doses are reported as milligrams of TPL per body weight (mg/kg).

### Animals

Female Wistar rats were used (170-230 g, n=6-8 animals for group), obtained from the Núcleo de Pesquisas em Plantas Medicinais, Universidade Federal do Piauí. The animals were kept under controlled laboratory conditions (22±1°C, 12-h alternate light-dark cycles, food and water *ad libitum*) and were acclimatized in the laboratory at least 1 h before testing. The protocols were approved by the Ethics Committee on Animal Experimentation/UFPI, 82/14) and were carried out in accordance with the current guidelines for the care of laboratory animals and the ethical guidelines for investigation of experimental pain in conscious animals (18).

### CFA-induced inflammation

The method used was based on the description by Auh and Ro (19). Female rats received 50 μL of CFA (0.5 mg/mL of *Mycobacterium butyricum*; Difco^®^) in the plantar surface of the right hind paw. Control group animals (sham) were injected with the same volume of 0.9% saline in the same manner. Twenty-four hours after CFA injection, the rats were orally (*po*) treated with DCF (1.25, 2.5 and 5.0 mg/kg), TPL (3.125, 6.25, 12.5, and 25 mg/kg) or vehicle (2% Tween 80 in 0.9% saline). For the drug association protocol, the selected doses were 3.125 mg/kg TPL+1.25 mg/kg DCF (TPL/DCF), which alone did not increase pain threshold. The positive control group received DFC (5 mg/kg).

**Figure 2:**
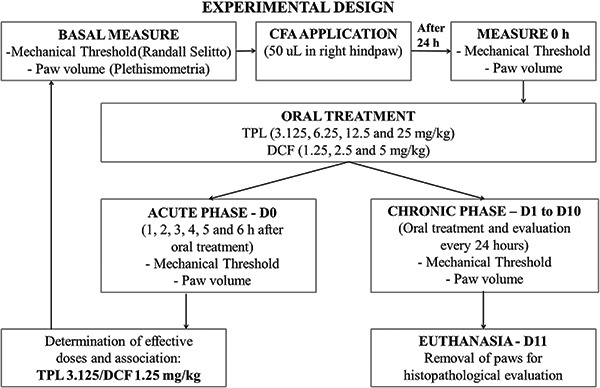
Experimental design. CFA: complete Freund's adjuvant; TPL: terpinolene; DCF: diclofenac.

Mechanical hyperalgesia and paw edema were examined immediately before (0) and (1, 2, 3, 4, 5 and 6 h) after treatment (acute phase). To investigate the effects of chronic treatment of TPL, animals were treated and 3 h later evaluated and tested once daily, during 10 days (chronic phase). The time when substances presented the best response was selected on day 0 (D0). Thus, during the next 10 days the substances were administered and the antihyperalgesic effect evaluated daily at this selected time (Figure 2).

### Mechanical hyperalgesia

The mechanical hyperalgesia was assessed by the Randall Selitto paw pressure test (20). Paw withdrawal thresholds were determined using an algesymeter (Insight^®^, Brazil). An increasing weight was applied to each paw until a withdrawal reflex was elicited. The investigator was trained to apply the tip perpendicular to the central area of the hind paw with a gradual increase in pressure. The end-point was characterized by the removal of the paw followed by clear movements. After paw withdrawal, the intensity of the pressure was recorded automatically. The frequency of withdrawal was determined before CFA injection (baseline), in order to obtain data purely derived from the treatments. The animals were tested before and after treatments. Three consecutive measurements were made at each time, and the thresholds were averaged for statistical analysis.

### Assessment of paw edema

The development of edema was quantified by measuring the volume of the hind paw using a digital plethysmometer (Insight^®^). Briefly, each paw was held in a liquid reservoir up to the level of the knee joint and the digital readout of volume displacement recorded. The results are reported in mL as the difference between paw volume before (baseline) and post-injection of CFA, which indicated the degree of edema.

### Leukocyte differential counts

On day 11, all female rats were sacrificed via anesthesia. Hind paws were removed for histological examination (n=5), immediately fixed by immersion in 10% formalin, and stored until examination. The tissues were processed using standard histological laboratory techniques. Briefly, using a microtome, 3-4 μm sections were cut, then stained with hematoxylin and eosin (H&E) stain following a standard staining protocol. The differential leukocyte counts were performed with the aid of optical microscopy (Olympus CX31, Japan) with oil immersion objective and 1.000× amplification. For each animal, 10 fields were randomly chosen, followed by the summation of the cells found (neutrophils, macrophages and lymphocytes) (21).

### Participation of the serotonergic system in the analgesic effect of terpinolene/diclofenac association (TPL/DCF)

To evaluate the role of serotonergic receptors in analgesic effect caused by the TPL/DCF association, inflammatory reaction was induced in the hind right paw of the animals with 50 µL of CFA. On the next day the animals were subcutaneously (*sc*) treated with ketanserin (3 mg/kg, 5-HT2A antagonist) or saline (22), and thirty minutes after, TPL/DCF, TPL (25 mg/kg), DCF (5 mg/kg) or vehicle (2% Tween 80 in saline 0.9%) were orally administered. After 1 h, the evaluation of the antihyperalgesic effect through paw compression test (Randall Selitto) in time intervals of 1, 2, 3, 4, 5 and 6 h was performed.

### Gastric evaluation

After rats were euthanized, the abdominal cavity was opened and the stomach removed for macroscopic evaluation and comparison of gastric lesions (23) between groups. Lesions were evaluated using the following scale: less than 10 petechiae: 2 points; 10 or more petechiae: 3 points; up to 1 mm ulcers: n × 2; ulcers larger than 1 mm: n × 3; perforated ulcers: n × 4, where n is the number of lesions observed; bleeding: 1 point; edema: 1 point; loss of folds: 1 point; loss of coloring: 1 point.

### Statistical analysis

Results are reported as mean±SEM. Statistical comparison of data was performed by two-way analysis of variance (ANOVA) followed by the Bonferroni's test or one-way ANOVA followed by the Tukey's test and Kruskal-Wallis test followed by Dunn's test. P<0.05 was considered to be significant (GraphPad Prism version 5.00 for Windows, GraphPad Software, USA; http://www.graphpad.com/).

## Results

### Effect of DCF on the CFA-induced mechanical hyperalgesia

**Figure 3:**
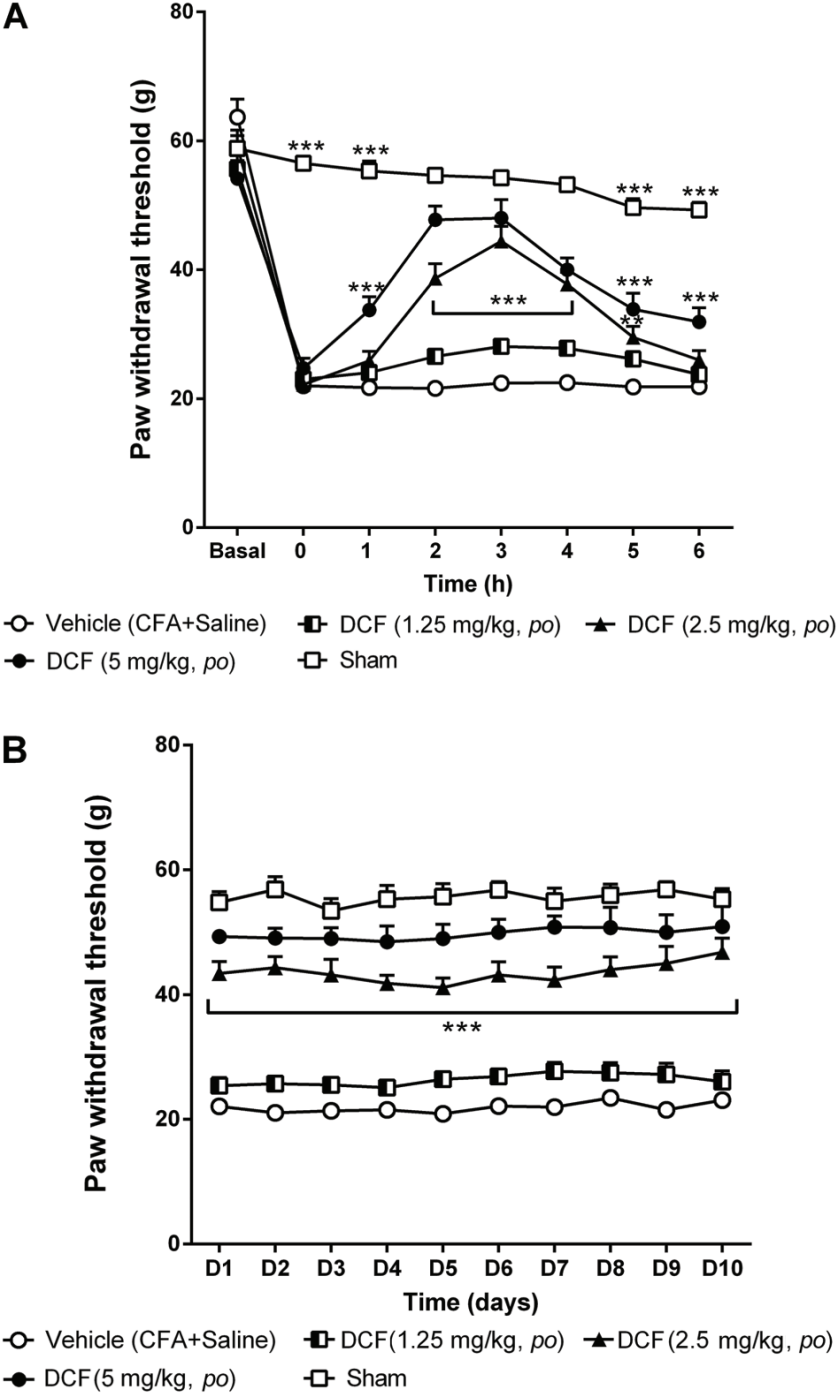
Effect of acute (*A*) and chronic (*B*) administration of diclofenac (DCF). Each point represents the mean±SEM of the paw withdrawal threshold in female Wistar rats (n=6-8) with mechanical hyperalgesia induced by Freund's complete adjuvant (CFA) (50 μL/paw) injection (*ipl*) subjected to the Randall Selitto test. **P<0.01 and ***P<0.001 compared to vehicle group (two-way ANOVA, Bonferroni test).

As can be seen in Figure 3, the paw withdrawal threshold decreased in all animals from the vehicle group 24 h after CFA injection, and this reduction was maintained during the whole experiment. Similarly, the lowest dose of DCF (1.25 mg/kg) had no analgesic effect on acute and chronic phases (Figure 3A and B, respectively). However, the 2.5 mg/kg dose significantly increased paw withdrawal threshold (44.44±2.31 g) from the second to the fifth hour of the acute phase (Figure 3A) and this analgesic effect returned from the first up to the tenth day of the chronic phase (46.82±2.24 g; Figure 3B; P<0.001). The highest dose of DCF (5 mg/kg) also significantly increased the pain threshold from the first to the sixth hour (Figure 3A), and this increase remained significant during the chronic phase (P<0.001) (Figure 3B).

### Effect of TPL on the CFA-induced mechanical hyperalgesia

**Figure 4:**
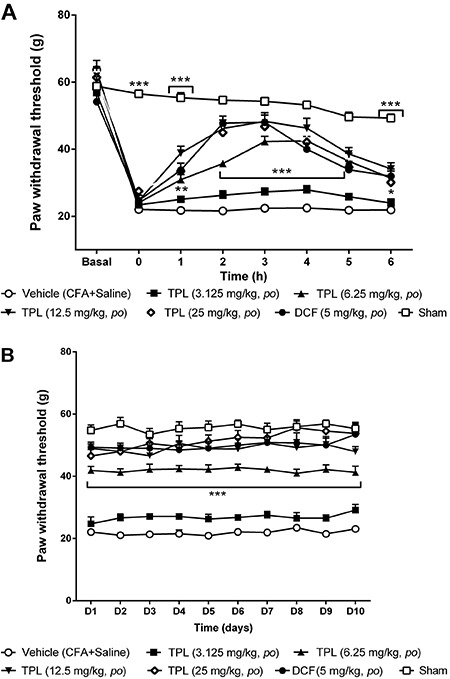
Effect of acute (*A*) and chronic (*B*) administration of terpinolene monoterpene (TPL). Each point represents the mean±SEM of the paw withdrawal threshold in female Wistar rats (n=6-8) with mechanical hyperalgesia induced by Freund's complete adjuvant (CFA) (50 μL/paw) injection (*ipl*) subjected to the Randall Selitto test. DCF: diclofenac. **P<0.01 and ***P<0.001 compared to vehicle group (two-way ANOVA, Bonferroni test).

The lowest dose of TPL (3.125 mg/kg) showed no antihyperalgesic effect in the acute and chronic phases, however, the 6.25, 12.5 and 25 mg/kg doses increased significantly the mechanical threshold from the second to the fifth hour (P<0.001) (acute phase) and these values were similar to the DCF 5 mg/kg, the positive control (Figure 4A). During the chronic phase (Figure 4B), the response was maintained during all the evaluation days, with similar values among the three tested doses (P<0.001; Figure 4).

### Effect of the TPL/DCF association on the CFA-induced mechanical hyperalgesia

**Figure 5:**
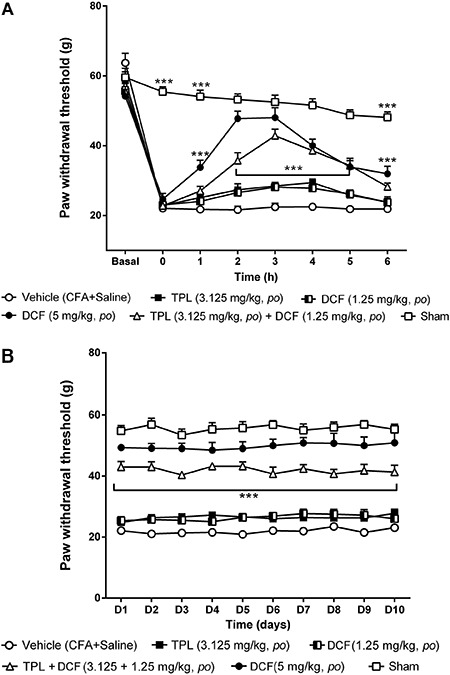
Effect of acute (*A*) and chronic (*B*) administration of terpinolene monoterpene (TPL)/diclofenac (DCF) association. Each point represents the mean±SEM of the paw withdrawal threshold in female Wistar rats (n=6-8) with mechanical hyperalgesia induced by Freund's complete adjuvant (CFA) (50 μL/paw) injection (*ipl*) subjected to the Randall Selitto test. ***P<0.001 compared to vehicle group (two-way ANOVA, Bonferroni test).

In Figure 5, the antinociceptive effect of the TPL/DCF association on the mechanical compression test is shown. TPL and DCF were used in ineffective doses (3.125 and 1.25 mg/kg, respectively), which did not significantly increase the mechanical threshold when substances were used alone. In the acute phase, the association TPL/DCF demonstrated a significant response from the second to fifth hour, similar to the positive control DCF (5 mg/kg). The comparison of these latter groups to the control group showed that TPL at 3.125 mg/kg increased mechanical threshold by 26.87% (P>0.05), DCF 1.25 mg/kg increased by 25.36% (P>0.05) and the combination caused a significant increase in the threshold of 91% (P<0.001) (Figure 5A). This effect remained significant for the duration of the chronic phase (P<0.001) (Figure 5B).

### Inhibitory effect of DCF on CFA-induced paw edema

**Figure 6:**
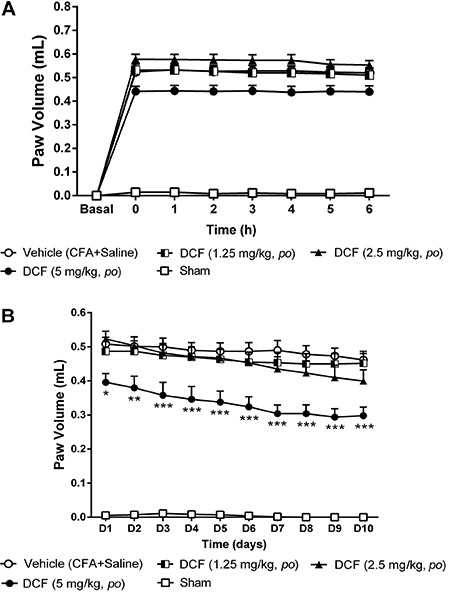
Effect of acute (*A*) and chronic (*B*) administration of diclofenac (DCF) on the paw edema induced by Freund's complete adjuvant (CFA) in female Wistar rats (n=6-8). Each point represents the mean±SEM of the paw volume (mL) before CFA injection (i.e., baseline) or at the times indicated thereafter. *P<0.05, **P<0.01 and ***P<0.001 compared to vehicle group (two-way ANOVA, Bonferroni test).

The local inflammatory process induced by CFA caused swelling in the paw of animals from the vehicle group, from the first hour until the tenth day of evaluation by plethismography. As can be seen in the Figure 6, DCF (1.25, 2.5 and 5 mg/kg) did not cause reduction of edema during the acute phase (Figure 6A). In the chronic phase, this NSAID (1.25 and 2.5 mg/kg) did not reduce paw edema, but DCF at 5 mg/kg decreased edema throughout the chronic phase (Figure 6B), from the first (D1) (P<0.05) to the tenth day (D10) (P<0.001) compared to the vehicle group.

### Inhibitory effect of TPL on CFA-induced paw edema

**Figure 7:**
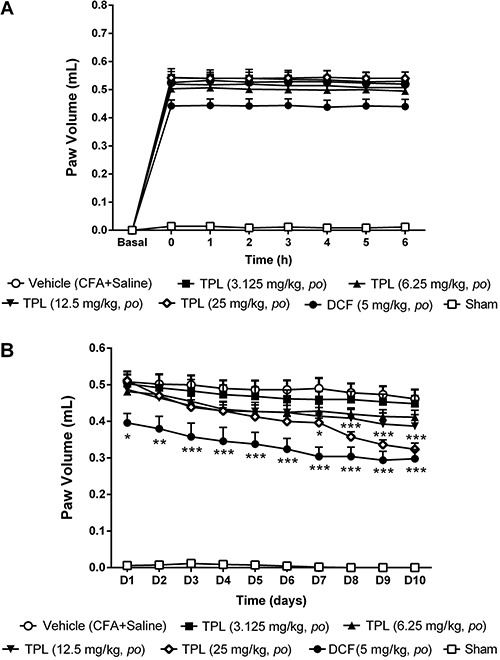
Effect of acute (*A*) and chronic (*B*) administration of terpinolene monoterpene (TPL) on the paw edema induced by Freund's complete adjuvant (CFA) in female rats (n=6-8). Each point represents the mean±SEM of the paw volume (mL) before CFA injection (i.e., baseline) or at the times indicated thereafter. DCF: diclofenac. *P<0.05, **P<0.01 and ***P<0.001 compared to vehicle group (two-way ANOVA, Bonferroni test).

TPL (3.125, 6.25, 12.5 and 25 mg/kg) caused no change in the volume of the animals paw during the acute phase (Figure 7A) compared to the vehicle group. During the chronic phase (Figure 7B), only the 25 mg/kg dose reduced paw edema in the last 4 days of evaluation. These values were 0.40±0.01 mL (D7, P<0.05); 0.36±0.01 mL (D8, P<0.001); 0.34±0.01 mL (D9, P<0.001) and 0.32±0.02 mL (D10, P<0.001). All values were significantly different compared to the vehicle group (0.49±0.02 mL) (Figure 7).

### Inhibitory effect of TPL/DCF association on CFA-induced paw edema

**Figure 8:**
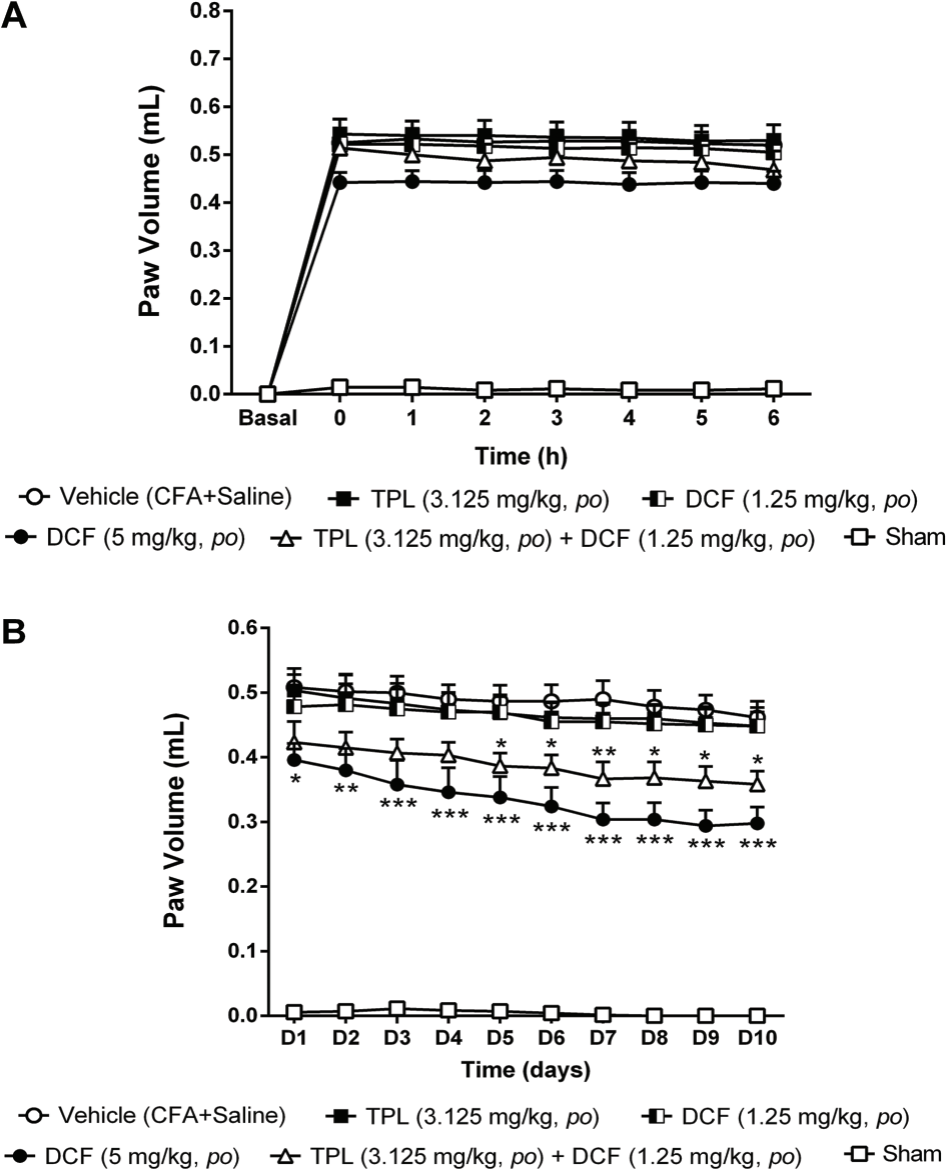
Effect of acute (*A*) and chronic (*B*) administration of terpinolene monoterpene (TPL)/diclofenac (DCF) association on the paw edema induced by CFA in female rats (n=6-8). Each point represents the mean±SEM of the paw volume (mL) before CFA injection (i.e., baseline) or at the times indicated thereafter. *P<0.05, **P<0.01 and ***P<0.001 compared to vehicle group (two-way ANOVA, Bonferroni test).

The TPL/DCF association did not change the volume of the animals’ paw in the acute phase (Figure 8A) when compared to the vehicle group. In the chronic phase (Figure 8B) the association reduced the paw edema from D5 to D10 when compared to the vehicle group. In the last five days of treatment, the TPL/DCF combination decreased paw edema by about 27% (Figure 8).

### Histopathological examination

**Figure 9:**
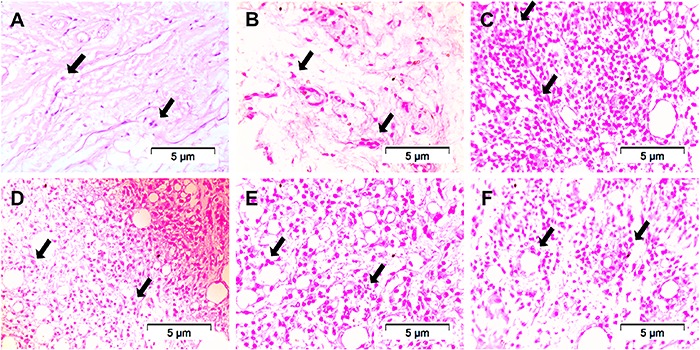
Histological analysis of the rat right hind footpad 11 days after injection of Freund's complete adjuvant (CFA) (40×) showing the number of immune cells (arrows). *A*, Contralateral paw *B*: Sham; *C*, vehicle; *D*, terpinolene monoterpene (TPL) (25 mg/kg, *po*); *E*, diclofenac (DCF) (5 mg/kg, *po*), and *F*, TPL (3.125 mg/kg, *po*)+DCF (1.25 mg/kg, *po*).

**Figure 10:**
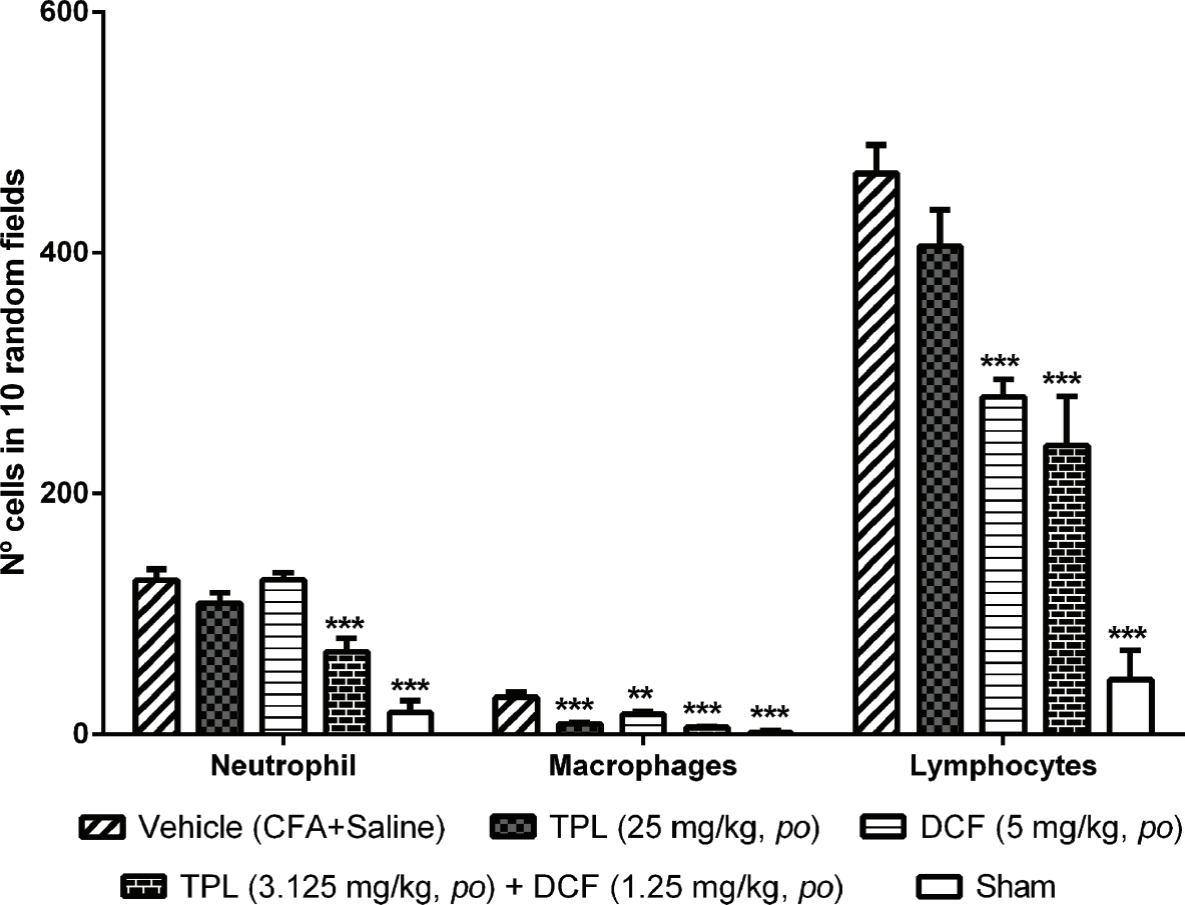
Differential leukocyte counts after 11 days of Freund's complete adjuvant (CFA) injection in the footpad of female Wistar rats (n=6). TPL: Terpinolene monoterpene; DCF: diclofenac. Data are reported as the mean±SEM of the number of cells/10 random fields. **P<0.01 and ***P<0.001 compared to vehicle group (one-way ANOVA, Tukey test).

The vehicle group presented a higher number of immune cells compared to the sham group and the contralateral paw. Chronic treatment with TPL (25 mg/kg), DCF (5 mg/kg), and TPL/DCF combination decreased the infiltration of these cells in the paw tissue (Figures 9 and 10). The group treated with TPL significantly reduced macrophages (8.60±1.21 cells/10 random fields, P<0.001) compared to the vehicle group. DCF decreased macrophages (16.40±2.50 cells; P<0.01) and lymphocytes (280.00±14.94 cells; P<0.001). The TPL/DCF combination reduced migration of the three types of cells: neutrophils by 46.48%, macrophages by 81.05% and lymphocytes by 48.50% (P<0.001).

### Participation of the serotonergic system in the antihyperalgesic effect of TPL/DCF association

**Figure 11:**
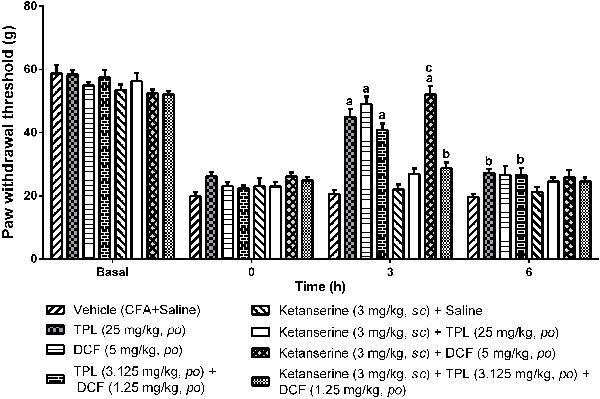
Involvement of the serotonergic system in the analgesic effect of terpinolene monoterpene (TPL)/diclofenac (DCF) combination in the mechanical compression test (Randall Selitto). Twenty-four hours after induction of inflammation by Freund's complete adjuvant (CFA) in female Wistar rats (n=6-8), the groups were treated with ketanserin (3 mg/kg *sc,* 5-HT2A antagonist) and vehicle, and then 30 min later, with TPL (25 mg/kg, *po*), TPL (3.125 mg/kg, *po*)+DCF (1.25 mg/kg, *po*) or DCF (5 mg/kg, *po*) as positive control. Data are reported as mean±SEM. ^a^P<0.001 and ^b^P<0.05 compared to saline group; ^c^P<0.001 compared to ketanserin + TPL group; ^d^P<0.001 compared to TPL+DCF group (two-way ANOVA, Bonferroni test).

In Figure 11, it can be seen that the 5HT-2A serotonin receptor antagonist ketanserin (KET) at 3 mg/kg reversed the antihyperalgesic effect of TPL (25 mg/kg). The group that received TPL increased the mechanical threshold from the first to the sixth hour (data not shown), being the highest value 45.00±2.5 g (third hour, P<0.001 compared to the vehicle group). The group receiving KET + TPL did not increase the threshold (3rd hour: 26.95±1.76 g and vehicle group: 20.55±1.25 g). KET did not reverse the antihyperalgesic effect of the DCF group (5 mg/kg) on the third hour (KET+DCF=49.17±2.30 g; DCF=52.08±2.53 g). On the other hand, KET reversed the antihyperalgesic effect of the TPL+DCF group (TPL+DCF=40.83±2.01 g; KET+TPL+DCF=8.75±1.81 g, P<0.05).

### Gastric macroscopic evaluation

Macroscopic analysis results showed that the use of TPL (3.125, 6.25, 12.5 and 25 mg·kg^-1^·day^-1^) and TPL+ DCF in female rats for 11 days did not cause gastric lesions, such as hyperemia or bleeding, compared to the sham group. The animals treated with DCF (1.25 and 2.5 mg/kg) showed the same results, while the animals that received DCF (5 mg·kg^-1^·day^-1^) showed a higher gastric lesion index compared to the vehicle (P<0.01; Table 1).

**Table:**
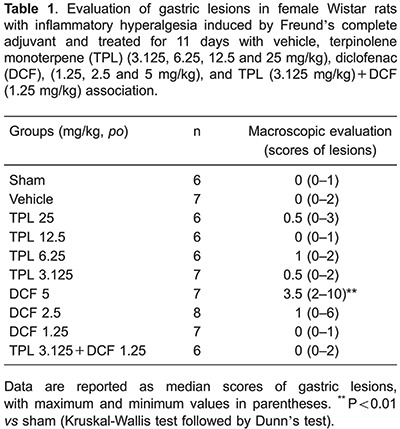

## Discussion

The monoterpene TPL presented antihyperalgesic effect in CFA-induced inflammatory pain in the acute and chronic phases. This effect seems to be mediated by the serotonin type-2A receptor (5-HT2A), since pretreatment with KET reversed the TPL antinociceptive effect. This result corroborates with another study that showed such reversion with the monoterpene citral (24).

After highlighting the effect of TPL on the inflammatory hyperalgesia, the TPL and DCF ineffective doses were combined and tested on the mechanical compression test to evaluate the acute and chronic phases. Interestingly, the antinociceptive effect of the combination was similar to the effective dose of DCF (5 mg/kg), which suggests a synergistic effect. Similar synergism was found between DCF and curcumin, in another study (25).

DCF presented pharmacokinetic parameters in accordance to the literature, as it reached maximum antinociceptive response at the third hour of acute assessment (26), and this response was maintained throughout the chronic phase.

We investigated if the DCF antinociceptive effect could be via serotonin-dependent mechanism, but only one 5-HT2A serotonin receptor antagonist (KET) was used, which did not reverse the effect of DCF. KET was used because its reversing effect on the acute antinociceptive effect of the natural substance TPL has already been demonstrated by our research group (data not published). This study suggests that one possible mechanism of action responsible for the antihyperalgesic effect of TPL/DCF is the synergistic effect on the central nervous system (CNS) via serotonergic system involvement, which was demonstrated by the pretreatment with KET, completely reversing this effect when compared with the TPL/DCF group.

The antiedematogenic effect of TPL, DCF and TPL/DCF on paw edema triggered by local application of CFA was also investigated (27). In the acute phase, none of the substances was able to significantly reduce paw edema. In the chronic phase, TPL, DCF and the TPL/DCF association presented antiedematogenic effects. The fact that DCF did not present an antiedematogenic effect in the acute phase, may be explained by the increase in COX-2 expression, following the administration of this NSAID (28). The DCF antiedematogenic effect in the chronic phase, can be attributed mostly to the inhibition of vasodilation and exudation mediated by the action of prostaglandins (29). Another possible action of DCF in reducing paw edema is the reduction of TNF-α and IL-1β cytokines (30). In this study, the treatment with DCF also caused a decrease in lymphocytes and macro-phages numbers in the paw tissue. There is previous evidence that both TNF-α and IL-1β present a critical role in the induction and perpetuation of the immune inflammation through activation of T lymphocytes and macrophages (31).

TPL presented antiedematogenic effect only in the last days of the chronic evaluation and at its highest dose. To date, no other study was found about TPL anti-inflammatory activity. In the same inflammatory pain model, TPL showed a result similar to that of linalool, a monoterpene with several biological activities, and this effect seems to be mediated by IL-1β and TNF-α cytokines (32). The histopathological analysis results showed a reduction of macrophages compared to the group that received saline.

The anti-inflammatory evaluation of TPL/DCF showed a stronger effect in the reduction of edema. This result corroborates a study that demonstrates the anti-inflammatory synergistic effect of the NSAID naproxen with the citral monoterpene in the carrageenan-induced paw edema, in which such effect was obtained with a combination of ineffective doses (8).

The TPL/DCF association also caused the reduction of neutrophils, macrophages and lymphocytes infiltration in the inflamed tissue. The effects of other TPL related monoterpenes or their associations to other drugs on leukocyte cell infiltration into the inflamed site has been found in the literature. In a peritonitis animal model, thymol, a monoterpene, had an anti-inflammatory effect, reducing the influx of leukocytes to the injured area (33).

The gastric toxicity was also investigated in our study, as the natural origin of the essential oils do not imply health safety. In the macroscopic analysis of the stomachs, only the group that received DCF showed macroscopic gastric lesions, while the TPL and TPL/DCF groups did not. There are no studies on the action of TPL in the gastric mucosa, but other monoterpenes have shown gastroprotective action. One of these examples is limonene, which was effective against lesions induced by pure ethanol and NSAIDs (34).

In summary, the combination of ineffective doses of TPL/DCF presented an important antihyperalgesic and anti-inflammatory effect against the CFA-induced chronic inflammation model. It was also evident that one of the possible mechanisms involved in this effect was mediated by serotonin receptors. These data suggest that the association of clinical drugs with natural products might be a pharmacological alternative in clinical use, avoiding expected adverse reactions. Thus, this preliminary study provides valuable information for the future development of a drug for chronic pain treatment.
